# Human Health Assessment of Sixteen Priority Polycyclic Aromatic Hydrocarbons in Contaminated Soils of Northwestern Algeria

**DOI:** 10.5696/2156-9614-11.31.210914

**Published:** 2021-08-17

**Authors:** Ahmed Halfadji, Mohamed Naous, Farida Bettiche, Abdelkrim Touabet

**Affiliations:** 1 Department of Sciences and Technology, Faculty of Applied Sciences, Ibn-Khaldoun University of Tiaret, Algeria.; 2 Synthesis and Catalysis Laboratory, Ibn-Khaldoun University of Tiaret, Algeria.; 3 Laboratory of Functional Organic Analysis, Faculty of Chemistry, Houari Boumediene University of Sciences and Technology, Algiers, Algeria.; 4 Laboratory of Macromolecular Physical Chemistry, Department of Chemistry, University Oran 1 Ahmed Ben Bella, Algeria; 5 Scientific and Technical Research Centre on Arid Regions, Campus Universitaire El Alia Nord, Biskra, Algeria

**Keywords:** Algerian public health, health risk assessment, incremental lifetime cancer risk, ILCR, polycyclic aromatic hydrocarbons, PAHs, soil pollution, total cancer risk

## Abstract

**Background.:**

Polycyclic aromatic hydrocarbons (PAHs) are ubiquitous environmental organic contaminants generated by incomplete combustion of organic materials that are widely distributed in soils.

**Objectives.:**

This study represents the first attempt to examine the health toxicity of 16 detected PAHs in contaminated soil, via different exposure pathways to populations in northwestern Algeria.

**Methods.:**

The toxicity equivalency quotients (TEQ) of PAHs were evaluated. The carcinogenic risk assessment of incremental lifetime cancer risk (ILCR) from ingestion, inhalation, and dermal exposure pathways to each PAH in soil are described.

**Results.:**

Incremental lifetime cancer risk values were in the upper limit of the tolerable range (10^−6^–10^−4^) for adults and children. The total cancer risk of PAH-contaminated soils for children, adolescents and adults was 2.48×10^−5^, 2.04×10^−5^ and 3.12×10^−5^mg.kg^−1^d^−1^, respectively. The highest potential cancer risks were identified for adults and children, with adolescents having the lowest risks. Across exposure pathways, the dermal contact and ingestion pathways had the greatest contributions to the carcinogenic risk of human exposure to PAHs.

**Conclusions.:**

Further research and guidelines are needed for risk assessments of PAHs in agricultural, residential/urban, and industrial areas, and further risk assessments should include risks posed by exposure through air.

**Competing Interests.:**

The authors declare no competing financial interests.

## Introduction

Polycyclic aromatic hydrocarbons (PAHs) are a class of organic molecules that contain more than two fused aromatic benzene rings.^1^

The majority of PAHs present in the environment are due to incomplete combustion and pyrolysis of organic matter.^2^,^3^ Recently, toxicity studies of 16 PAHs listed as priority pollutants by the United States Environmental Protection Agency (USEPA) have received considerable attention.^4^–^6^ These PAHs are of particular interest due to their mutagenic activities as some are classified as probable human carcinogens and others are potentially carcinogenic.^7^ Furthermore, benzo[a]pyrene (BaP) has been characterized as having a local and systemic carcinogenic effect, as well as widespread persistence in diverse environmental matrices such as soil, dust, sediments, and water due to its lipophilic and hydrophobic properties.^8^ The International Agency for Research on Cancer (IARC) classified benzo(a)pyrene as carcinogenic to humans, along with seven PAHs: Group I, benzo(a)anthracene, dibenzo(a,l)pyrene, and dibenzo(a,h)anthracene as probable carcinogens; and Group II, naphthalene, chrysene, benzo(b)fluoranthene, benzo(j)fluoranthene, benzo(k)fluoranthene and indeno(1,2,3-c,d)pyrene as possible carcinogens.^9^ These PAHs have been shown to have toxic effects on human reproductive, developmental, cardiorespiratory, and immune systems.^9^ Most of the questions about the toxicological effects of PAHs on humans are related to their carcinogenesis, mutagenesis, and endocrine disruption.^10^ Furthermore, PAH toxicity may have estrogen and antiestrogen effects, which may influence the risk of breast cancer, stomach cancer, dermatitis, gastroenteritis, and pneumonia.^11^,^12^ The soil system is considered to be a good reservoir of organic contaminants, including PAH compounds, owing to their ubiquity, stability, and long-term enrichment in soils. A high concentration of PAHs has been found in many surface soils around the world, among the numerous environmental matrices (water, dust, soil, plant, and sediments).^13^ However, PAH-contaminated surface soil can directly or indirectly expose humans, so it is critical to assess the risk of PAH-contaminated soils to protect human health.^9^ Health and cancer risk evaluations focused on various methods of specific exposure criteria and age ranges have been extensively described for populations in various locations around the world, but there are only a few cases of studies recorded for the African community in this context.^10^,^14^–^17^ It is therefore necessary to assess the potentially harmful effects of PAHs in soils in order to inform future remediation decisions.^18^

Polyaromatic hydrocarbons are widespread in the studied area of northwestern Algeria as a result of industrial, commercial and transportation activities, and cereal cultivation.^19^,^20^

This research represents the first evaluation of the cancer risk for three population groups: children (2 to 12 years old), adolescents (13 to 20 years old), and adults (over 20 years old) who were exposed to 16 PAHs through contaminated surface soils via three different exposure pathways: ingestion, inhalation, and dermal contact in northwestern Algeria.

**Abbreviations**
*BaPeq*: BaP toxic equivalent concentration
*CDI*: Chronic daily intake
*EC*: Toxic equivalent quotient
*USEPA*: United States Environmental Protection Agency

## Methods

**Figure 1:**
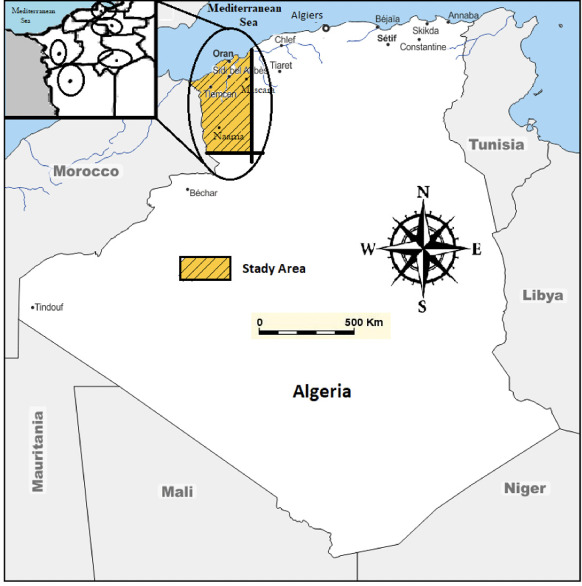
Map of study area showing sampling points and population concentration in Northwestern Algeria

The population of Algeria's northwestern region is concentrated in five cities (Tlemcen, Sidi Bel Abbes, Ain Temouchent, Mascara, and Oran) *(Figure 1),* which covers an area of 33 030 km^2^ and have a density of 1 582 818 inhabitants. With a median age of about 28.5 years, this population accounts for 3.65% of all Algerian residents.^21^ The study area has a Mediterranean climate with moderate, wet winters and warm, dry summers, with annual average temperatures ranging from 43°F to 86°F, annual rainfall ranging from 400 to 670 mm, and an average hourly wind speed of 8.4 to 11.1 miles per hour.^22^ In the study area, PAH-contaminated surface soil samples were localized in several locations from different fields: industrial park area surrounding cement factories, as well as urban/rural, residential, cereal agricultural and industrial zones.^19^,^20^

### Polyaromatic hydrocarbons in soil

Previous studies and published results in this area of northwestern Algeria have reported monitoring data on 16 individual PAHs in surface soils, as seen in Table 1. The concentration of Σ20 PAHs identified in surface soils from industrial cement, urban, cereal agricultural, and rural sites were 10 724, 741, 385, and 246 μg/kg, respectively.^19^ In addition, concentrations for the Σ16 PAHs ranged from 188.07–2068.04, 246.86–1918.0, and 133.72–249.69 μg/kg for industrial, urban, and agricultural soils, respectively.^20^ The concentrations of the 16 PAHs detected in surface soil samples from all regions were converted into maximum, minimum, and means concentrations in the present investigation. The present study calculated the health risk to the population in this area from inhalation, ingestion, and dermal contact exposure to soils polluted with 16 PAHs based on these data *(Table 1).*

**Table 1:** Benzo[a]pyrene Toxic Equivalency (BaPeq) and Concentrations of 16 PAHs (μg/kg) Detected in Soil Samples of the Study Area

	Levels of 16 PAHs in sampling sites * (μg/kg) (2012)					Levels of 16 PAHs in surface soils of study area (μg/kg) (2018)			Levels of 16 PAHs in surface soils of study area (μg/kg]			BaPeq (μg Teq/kg)		
PAH	A	TEF^23^	Mean * μg/kg	*Min*	*Max*	Mean ** μg/kg	*Min*	*Max*	*Mean μg/kg*	*Min*	*Max*	*Mean μg/kg*	*Min*	*Max*
Naphthalene	2	0.001	36.16	19.90	64.18	0.16	0.04	0.36	18.16	0.04	64.18	0.02	4.18 × 10^−5^	0.06
Acenaphthylene	3	0.001	17.06	3.94	32.63	33.93	0.02	133.37	25.50	0.02	133.37	0.03	1.93 × 10^−5^	0.13
Acenaphthene	3	0.001	9.38	4.14	16.32	0.84	0.02	2.25	5.11	0.02	16.32	0.01	1.91 × 10^−5^	0.02
Fluorene	3	0.001	8.45	4.13	13.56	1.80	0.75	3.63	5.13	0.75	13.56	0.01	7.51 × 10^−4^	0.01
Phenanthrene	3	0.001	57.43	19.25	110.49	131.64	36.73	399.09	94.53	19.25	399.09	0.09	0.02	0.40
Anthracene	3	0.01	12.05	2.88	24.21	65.88	1.49	251.37	38.97	1.49	251.37	0.39	0.01	2.51
Fluoranthene	4	0.001	90.49	19.37	176.49	687.18	33.03	2513.46	388.84	19.37	2513.46	0.39	0.02	2.51
Pyrene	4	0.001	80.49	16.47	158.75	697.50	132.56	2342.65	388.99	16.47	2342.65	0.39	0.02	2.34
Benzo[a] anthracene	4	0.1	52.71	8.98	90.11	515.09	5.11	1920.02	283.90	5.11	1920.02	28.39	0.51	192.00
Chrysene	4	0.01	23.71	7.60	42.31	137.70	4.43	482.93	80.71	4.43	482.93	0.81	0.04	4.83
Benzo[b] fluoranthene	5	0.1	124.92	21.59	191.02	198.22	3.22	720.57	161.57	3.22	720.57	16.16	0.32	72.06
Benzo[k] fluoranthene	5	0.1	55.29	10.12	88.86	116.98	1.86	421.56	86.14	1.86	421.56	8.61	0.19	42.16
Benzo[a]pyrene (BaP]	5	1	76.39	16.35	121.23	140.09	2.19	506.48	108.24	2.19	506.48	108.24	2.19	506.48
Indeno(1,2,3-c,d)pyrene	6	0.01	77.86	8.51	133.27	97.84	2.83	345.67	87.85	2.83	345.67	0.88	0.03	3.46
Dibenzo(a,h)anthracene	5	5	19.95	2.81	34.97	23.04	1.20	81.33	21.50	1.20	81.33	107.48	10.20	181.33
Benzo (g,h,i]perylene	6	0.1	87.25	14.73	140.74	80.74	2.55	280.34	84.00	2.55	280.34	8.40	0.26	28.03
Σ16 PAHs			829.6	180.8	1350.3	2928.6	239.8	10404.8	1879.11	180.77	10404.81			
BaPeqΣ_7_PAHs_Carcin_												270.57 (96.5%)	13.48	1002.32
TEQ												280.28	14.81	1038.35

Abbreviations: A, number of aromatic rings; TEF, toxic equivalency factor; TEQ, toxic equivalency quotient

^*^16 PAHs reported in previously study, Halfadji *et al*.^20^

^**^ 16 PAHs from 20 PAHs reported in study area, Djellouli *et al.*^19^

#### Toxic equivalent concentrations

Benzo[a]pyrene is one of the most potent carcinogenic PAHs.^12^ In the present study, the toxicities of PAHs of contaminated surface soil samples were evaluated by calculating the toxicity value of each PAH based on the set of toxicity equivalency factors (TEFs) developed by Nisbet and lagoy (1992) *(Table 1).*^23^ The total BaP equivalent concentration (BaPeq) was calculated by the sum of BaPeq for each PAH using toxicity equivalent factors, and the toxic equivalent quotient (TEQ) of soil was calculated by collecting the products of each individual PAH content, following Equations 1 and 2,^23^,^24^:

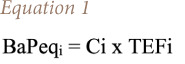


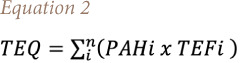
where, BaPeq is a toxic equivalent concentration, Ci is a concentration of PAHi, and TEFi is a toxicity equivalence factor for each PAH.

### Risk assessment of polyaromatic hydrocarbon models

In this research, chronic daily intakes (CDIs) associated with PAH exposure in the soil through the three pathways (ingestion, inhalation, and dermal contact) were evaluated by the formulae reported by the USEPA (1991)^25^, following Equation 3–5:

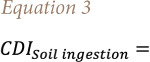

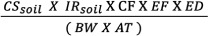


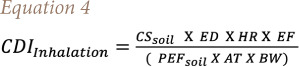


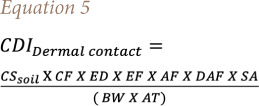
where CDI_ingestion_ is the chronic daily intake related to soil particle ingestion (mg/kg/day). The CDI of PAHs in other mediums was not considered as this study only addresses the PAHs carried by soil dust particles. CS_soil_ is the concentration of PAHs in soil (mg/kg), IRsoil is the ingestion rate of soils (mg/day), EF is the exposure frequency (d. year^−1^), ED denotes exposure duration (years), BW is the bodyweight of the exposed individual (kg),^26^,^27^ LT is a lifetime (WHO 2006), AT is the average time (days) for lifetime exposure of cancer risk (AT=LTx365), CF is a conversion factor (1. 10^−6^ kg/mg), CDI_inhalation_ is the chronic daily intake via inhalation of soil particles (mg kg^−1^ d^−1^), HR is the air inhalation rate (m^3^/d), PEF_soil_ is the soil particle emission factor, CDI_dermal contact_ is the chronic daily intake for dermal contact of soil (mg/kg/d), SA is the skin surface area available for contact soil (cm^2^/day), AF is the relative skin adherence factor for soil (mg/cm^−2^), and ABS is the dermal absorption fraction. The values of all variables are reported in Table 2.

**Table 2:** Exposure Variables for the Health Risk Assessment for Children, Adolescents and Adults via Ingestion, Inhalation and Dermal Exposure Pathways

**Definition**	**Units**	**Children 2–12 years**	**Adolescents 13–-20 years**	**Adults >20 years**	**Reference**
Average body weight	Kg	24.2	56.8	69.6	26
Unit conversion factor	Kg/mg	1.00×10^6^	1.00×10^6^	1.00×10^6^	25, 28
Exposure frequency	Days/years	350	350	350	25
Exposure duration	Years	7	10	41	28
Inhalation rate	m^3^/day	10	16	16	53,28
Soil ingestion rate	mg/day	200	100	100	25
Dermal surface exposure	cm^2^/day	2800	5700	5700	53
Dermal adherence factor	mg /cm^2^	0.2	0.2	0.07	53
Dermal adsorption fraction	Unitless	0,13	0,13	0,13	53
Lifetime	Years	72	72	72	25
Average life span	Days	26 280	26 280	26 280	53
Particulate emission factor	m^3^/kg	1.36×10^9^	1.36×10^9^	1.36×10^9^	53
Cancer slope factor ingestion	mg/kg/day	7.3	7.3	7.3	29
Cancer slope factor dermal	mg/kg/day	25	25	25	29
Cancer slope factor inhalation	mg/kg/day	3.85	3.85	3.85	29

### Human health risk assessment

According to the USEPA's Exposure Factor Handbook,^28^ the incremental lifetime cancer risk (ILCR) model was used to evaluate the risk to residents exposed to PAHs in soil. The ILCRs for each of the three contact pathways resulting from intake of daily contact products (CDIs) to oral slope factor cancer (CSFs) is shown in Equation 6:

ILCR = CDI × Cancer oral slope factor (CSF)


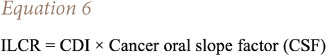


Where CSF is the carcinogenic slope factor (mg/kg/day). CSF_ingestion_, CSF_dermal_, and CSF_inhalation_ of BaP were considered to be 7.3, 25, and 3.85 (mg/kg/day)^−1^, respectively, based on the cancer-causing ability of BaP.^29^,^30^

R represents the total risk of cancer resulting from the sum of the ILRC associated with each exposure contact as follows in Equation 7:

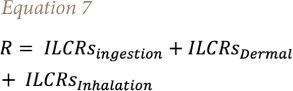



Where Cs is the PAH concentration of soil samples (mg/kg), other variables are shown in Table 1, and the cancer risks for children (2 to 12 years), adolescents (13 to 20 years), and adults ( >20 years) were calculated separately.

According to the USEPA and regulatory programs, an ILCR of ≤10^−6^ denotes the level of risk considered acceptable or inconsequential, an ILCR of ≥10^−4^ is considered a serious risk requiring high priority attention, and an ILCR from < 10^−6^ to <10^−4^ reflects a potential risk to human health.^28^,^31^–^33^ A cancer risk (R) value greater than 10^−6^ is considered unacceptable according to the USEPA.^34^,^35^

## Results

Only benzo(a)pyrene, from of the 16 PAHs considered in the present study, has toxicity data that can be used to calculate the carcinogenicity factor. The carcinogenic risk due to exposure to each PAH was estimated by the toxicity equivalent of BaP (BaPeq).^36^ In the present study, the toxicity of PAHs in soil samples from northwest Algeria was evaluated using the TEQs of 16 PAHs and the BaPeq was computed from the soil sample based on the TEF values^23^. The BaPeq and concentrations of 16 PAHs (μg/kg) detected in soil samples are shown in Table 1.

The human health risk is assessed based on possible exposures to PAH-contaminated soil surfaces, either directly or indirectly, and according to various parameters for children, adolescents, and adults such as exposure frequency, exposure duration, body weight, and average life span *(Table 2).* Intake daily contacts (CDIs) and carcinogenic risks (ILCRs) for each PAH for three age groups (children, adolescents, adults) and exposure pathways (inhalation, ingestion, dermal contact) in the study area were evaluated based on Equation 2 to 6 and the results are reported in Tables 3–5 and Supplemental Material Table S2.

**Table 3:** Incremental Lifetime Cancer Risk (ILRC) for Human Exposure to Individual Polyaromatic Hydrocarbons (PAHs) via Dermal Contact Exposure to Surface Soils in the Study Area

**Individual PAH**	**ILCR Carcinogenic risk Dermal contact**		

	**Children**	**Adolesents**	**Adults**
Naphthalene	1.33 × 10^−7^	1.65 × 10^−7^	1.93 × 10^−7^
Acenaphthylene	1.86 × 10^−7^	2.31 × 10^−7^	2.70 × 10^−7^
Acenaphthene	3.74 × 10^−8^	4.63 × 10^−8^	5.42 × 10^−8^
Fluorene	3.75 × 10^−8^	4.64 × 10^−8^	5.44 × 10^−8^
Phenanthrene	6.91 × 10^−7^	8.56 × 10^−7^	1.00 × 10^−6^
Anthracene	2.85 × 10^−7^	3.53 × 10^−7^	4.13 × 10^−7^
Fluoranthene	2.84 × 10^−6^	3.52 × 10^−6^	4.13 × 10^−6^
Pyrene	2.84 × 10^−6^	3.52 × 10^−6^	4.13 × 10^−6^
Benzo[a]anthracene	2.08 × 10^−6^	2.57 × 10^−6^	3.01 × 10^−6^
Chrysene	5.90 × 10^−7^	7.31 × 10^−7^	8.56 × 10^−7^
Benzo[b ]fluoranthene	1.18 × 10^−6^	1.46 × 10^−6^	1.71 × 10^−6^
Benzo[k]fluoranthene	6.30 × 10^−7^	7.80 × 10^−7^	9.14 × 10^−7^
Benzo[a]pyrene (BaP)	7.91 × 10^−7^	9.81 × 10^−7^	1.15 × 10^−6^
Indeno(1,2,3-c,d)pyrene	6.42 × 10^−7^	7.96 × 10^−7^	9.32 × 10^−7^
Dibenzo(a,h)anthracene	1.57 × 10^−7^	1.95 × 10^−7^	2.28 × 10^−7^
Benzo(g,h,i)perylene	6.14 × 10^−7^	7.61 × 10^−7^	8.91 × 10^−7^
Σ2–3-ring	1.37 × 10^−6^	1.70 × 10^−6^	1.99 × 10^−6^
Σ4ring	8.35 × 10^−6^	1.04 × 10^−5^	1.21 × 10^−5^
Σ5ring	2.76 × 10^−6^	3.42 × 10^−6^	4.00 × 10^−6^
Σ6ring	1.26 × 10^−6^	1.56 × 10^−6^	1.82 × 10^−6^
**Σ*16 PAHs***	**1.37 × 10^−5^**	**1.70 × 10^−5^**	**1.99 × 10^−5^**

**Table 4—:** Incremental Lifetime Cancer Risk (ILRC) for Human Exposure to Individual Polyaromatic Hydrocarbons (PAHs) via Ingestion to Surface Soils in the Study Area

**Individual PAH**	**ILCR Carcinogenic risk Ingestion**		

	**Children**	**Adolescents**	**Adults**
Naphthalene	1.07 × 10^−7^	3.24 × 10^−8^	1.08 × 10^−7^
Acenaphthylene	1.50 × 10^−7^	4.55 × 10^−8^	1.52 × 10^−7^
Acenaphthene	3.00 × 10^−8^	9.12 × 10^−9^	3.05 × 10^−8^
Fluorene	3.01 × 10^−8^	9.15 × 10^−9^	3.06 × 10^−8^
Phenanthrene	5.54 × 10^−7^	1.69 × 10^−7^	5.65 × 10^−7^
Anthracene	2.29 × 10^−7^	6.96 × 10^−8^	2.33 × 10^−7^
Fluoranthene	2,28 × 10^−6^	6.94 × 10^−7^	2.32 × 10^−6^
Pyrene	2,28 × 10^−6^	6.94 × 10^−7^	2.32 × 10^−6^
Benzo[a]anthracene	1.67 × 10^−6^	5.07 × 10^−7^	1.70 × 10^−6^
Chrysene	4.73 × 10^−7^	1.44 × 10^−7^	4.82 × 10^−7^
Benzo[b]fluoranthene	9.48 × 10^−7^	2.88 × 10^−7^	9.65 × 10^−7^
Benzo[k]fluoranthene	5.05 × 10^−7^	1.54 × 10^−7^	5.14 × 10^−7^
Benzo[a]pyrene (BaP)	6.35 × 10^−7^	1.93 × 10^−7^	6.46 × 10^−7^
Indeno(1,2,3-c,d)pyrene	5.15 × 10 ^7^	1.57 × 10^−7^	5.25 × 10^−7^
Dibenzo(a,h)anthracene	1.26 × 10^−7^	3.84 × 10^−8^	1.28 × 10^−7^
Benzo(g,h,i)perylene	4.93 × 10^−7^	1.50 × 10^−7^	5.02 × 10^−7^
Σ2–3-ring	1.10 × 10^−6^	3.34 × 10^−7^	1.12 × 10^−6^
Σ4ring	6.70 × 10^−6^	2.04 × 10^−6^	6.82 × 10^−6^
Σ5ring	2.21 × 10^−6^	6.74 × 10^−7^	2.25 × 10^−6^
Σ6ring	1.01 × 10^−6^	3.07 × 10^−7^	1.03 × 10^−6^
**Σ*16 PAHs***	**1.10 × 10^−5^**	**3.35 × 10^−6^**	**1.12 × 10^−5^**

**Table 5:** Incremental Lifetime Cancer Risk (ILRC) for Human Exposure to Individual Polyaromatic Hydrocarbons (PAHs) via Inhalation Exposure to Surface Soils in the Study

**Individual PAH**	**ILCR Carcinogenic risk Dermal contact**		

	**Children**	**Adolescents**	**Adults**
Naphthalene	2.07 × 10^−12^	2.01 × 10^−12^	6.73 × 10^−12^
Acenaphthylene	2.90 × 10^−12^	2.82 × 10^−12^	9.45 × 10^−12^
Acenaphthene	5.81 × 10^−13^	5.66 × 10^−13^	1.89 × 10^−12^
Fluorene	5.83 × 10^−13^	5.68 × 10^−13^	1.90 × 10^−12^
Phenanthrene	1.08 × 10^−11^	1.05 × 10^−11^	3.50 × 10^−11^
Anthracene	4.43 × 10^−12^	4.32 × 10^−12^	1.44 × 10^−11^
Fluoranthene	4.42 × 10^−11^	4.31 × 10^−11^	1.44 × 10^−10^
Pyrene	4.42 × 10^−11^	4.31 × 10^−11^	1.44 × 10^−10^
Benzo[a]anthracene	3.23 × 10^−11^	3.14 × 10^−11^	1.05 × 10^−10^
Chrysene	9.18 × 10^−12^	8.94 × 10^−12^	2.99 × 10^−11^
Benzo[b]fluoranthene	1.84 × 10^−11^	1.79 × 10^−11^	5.99 × 10^−11^
Benzo[k]fluoranthene	9.80 × 10^−12^	9.54 × 10^−12^	3.19 × 10^−11^
Benzo[a]pyrene (BaP)	1.23 × 10^−11^	1.20 × 10^−11^	4.01 × 10^−11^
Indeno(1,2,3-c,d)pyrene	9.99 × 10^−12^	9.73 × 10^−12^	3.26 × 10^−11^
Dibenzo(a,h)anthracene	2.44 × 10^−12^	2.38 × 10^−12^	7.97 × 10^−12^
Benzo(g,h,i)perylene	9.55 × 10^−12^	9.30 × 10^−12^	3.11 × 10^−11^
Σ2–3-ring	2.13 × 10^−11^	2.08 × 10^−u^	6.94 × 10^−11^
Σ4ring	1.30 × 10^−10^	1.27 × 10^−10^	4.23 × 10^−10^
Σ5ring	4.29 × 10^−11^	4.18 × 10^−11^	1.40 × 10^−10^
Σ6ring	1.95 × 10^−11^	1.90 × 10^−11^	6.37 × 10^−11^
Σ***16 PAHs***	**2.14 × 10^−10^**	**2.08 × 10^−10^**	**6.96 × 10^−10^**

In the present assessment of human health exposure to PAH-contaminated soils, the ILCR was chosen to assess and diagnose the potential cancer risk (R) for different age groups via three pathway exposures, based on the CSF and CDI. The results of ILCRs and R values (min, max and mean) for three population groups: children (2–12 years of age), adolescents (13–20 years of age), and adults (>20 years of age), via different exposure pathways (inhalation, ingestion, and dermal contact) are presented in Tables 3–6.

**Table 6:** Cancer Risk and Corresponding Incremental Lifetime Cancer Risk Across Age Groups via Different Exposure Routes to Contaminated Soil

	**Children (2–12 years)**			
Exposure pathway	ILCR ingestion	ILCR inhalation	ILCR dermal	Cancer risk (R)
Min	4.74 × 10^−7^	9.19 × 10^−12^	5.91 × 10^−7^	1.06 × 10^−6^
Max	6.15 × 10^−5^	1.19 × 10^−9^	7.67 × 10^−5^	1.38 × 10^−4^
Mean	**1.10 × 10^−5^**	**2.14 × 10^−10^**	**1.37 × 10^−5^**	**2.48 × 10^−5^**
	**Adolescents (13–20 years)**			
Exposure pathway	ILCR ingestion	ILCR inhalation	ILCR dermal	R
Min	1.44 × 10^−7^	8.95 × 10^12^	7.32 × 10^−7^	8.76 × 10^−7^
Max	1.87 × 10^−5^	1.16 × 10^−9^	9.51 × 10^−5^	1.14 × 10^−4^
Mean	**3.35 × 10^−6^**	**2.08 × 10^−10^**	**1.70 × 10^−5^**	**2.04 × 10^−5^**
	**Adults (>20 years)**			
Exposure pathway	ILCR ingestion	ILCR inhalation	ILCR dermal	R
Min	4.83 × 10^−7^	2.99 × 10^−11^	8.57 × 10^−7^	1.34 × 10^−6^
Max	6.27 × 10^−5^	3.89 × 10^−9^	1.11 × 10^−4^	1.74 × 10^−4^
**Mean**	**1.12 × 10^−5^**	**6.96 × 10^−10^**	**1.99 × 10^−5^**	**3.12 × 10^−5^**

The average values of total ILCRs calculated for the study area indicate that risk levels via dermal contact for children, adolescents, and adults ranged from 1.37 × 10^−5^ to 1.99 × 10^−5^ *(Table 3),* while the risk levels of cancer via ingestion ranged from 3.35 × 10^−6^ to 1.12 × 10^−5^ *(Table 4)* and inhalation exposures ranged from 2.08 × 10^−10^ to 6.96 × 10^−10^ *(Table 5).* In addition, the cancer risk level for adults was 3.12 × 10^−5^, 2.48 × 10^−5^ for children, and 2.04 × 10^−5^ for adolescents (*Table 6*).

## Discussion

The present study revealed that BaPeq values for 16 PAHs in the study area averaged 280.28 g/kg, which is less than 300 g/kg and much lower than the Canadian Soil Quality Guideline recommendation of TEQ < 600 g/kg.^37^ However, the BaPeq levels for surface soils in the study area exceeded the Dutch target value of 33 g. kg^−1^.^38^ In addition, the TEQ values also reflect the relative carcinogenic potential of all 16 PAHs to BaP in soil samples *(Table 1).*

The contribution of BaPeq for each PAH to total TEQ estimates were in the following order: benzo[a]pyrene (BaP) (38.6%) > dibenzo(a,h)anthracene (38.3%) > benzo[a]anthracene > benzo[b]fluoranthene > benzo[k]fluoranthene > benzo(g,h,i) perylene > indeno(1,2,3-c,d)pyrene > chrysene > anthracene > fluoranthene > pyrene > phenanthrene> acenaphthylene > naphthalene > fluorene > acenaphthene (*Table 1*). These results show that BaP (38.6%) and dibenzo(a,h)anthracene (38.3%) were the highest contributors to the risk of carcinogenicity according to the TEQ, similar to the results reported in the Indian states of Assam and Chhattisgarh,^39^,^40^ and for reed wetland soils of Liaohe Estuary in China.^41^ Other studies have indicated that BaP has a similar influence on TEQ.^39^,^42^–^44^ This further confirms the importance of BaP as an indicator of overall PAH carcinogenicity. The average BaPeq value for the seven carcinogenic PAHs (BaPeqΣ7PAHs_Carcin_) contributes more than 96.5% of the total Σ16 BaPeq level in the present study, explaining why the seven carcinogenic PAHs are the most toxic of the 16 PAH total components.

The results of TEQ in northwestern Algerian surface soils *(Table 1)* varied between 14.81 to 1038.35 μg/kg, with an arithmetic mean of 280.28 μg/kg, which is lower than those in soils from regions of Kucming, Shenfu, and Beijing, China (100–3400 μg/kg)^45^,^46^ and Lagos, Nigeria (523–1046 μg/kg),^38^ but higher than reported in Delhi, India (4.39–717.06 μg/kg, mean of 131.46 μg/kg),^47^ Zhongyuan oilfield, (9.10–75.83 μg/kg),^48^ Beijing, China (39.4–559.5 μg/kg),^49^ and Isola Delle Femmine, Italy (3.3–69 μg/kg).^50^

### Health risk assessment and contribution of individual polyaromatic hydrocarbons

To date, no environmental guidelines have yet been established in Algeria for PAH-contaminated soils. According to most regulatory programs and guidelines around the world, an ILCR less than or equal to 10^−6^ denote a negligible risk, ILCRs of 10^−6^ to 10^−4^ indicate potential risk, and ILCRs greater than 10^−4^ indicate potential high risk.^25^,^51^,^52^

The chronic daily intakes (CDIs) of the sum of low molecular weight (LMW) *Σ_LMW_−*PAHs (5.39 × 10^−12^ – 1.53 × 10^−7^ mg/kg/d) for all age groups are less than those of high molecular weight (HMW) *Σ_HMW_−*PAHs (4.87 × 10^−11^ – 1.83 × 10^−6^ mg/kg/d) *(Supplemental Material).* The 4-ring PAHs and the 6-ring PAHs are the main contributors to BaPeq and increasing CDIs among the 16 PAHs.

The results of the ILCR carcinogenic risk of individual PAH surface soil *(Tables 3–5)* polluted with fluoranthene, pyrene, benzo[b]fluoranthene, and benzo[a]anthracene contributes substantially to the potential cancer risk (ILCR ≥ 10^−6^) of children and adults by ingestion and dermal contact exposures, and these compounds also contribute to potential cancer risk (ILCR ≥ 10^−6^) of adolescents via dermal contact. In the present study, the high molecular weight polycyclic aromatic hydrocarbons (HMW PAHs) with four rings contributed the most to the ILCRs for all age groups (*Table 3–5*). The 5- and 6-ring HMW PAHs such as benzo[a]pyrene (BaP), benzo[b]fluoranthene, benzo[k]fluoranthene, indeno(1,2,3-c,d)pyrene, and benzo(g,h,i)perylene have a moderate contribution to the cancer risk for adults and children. The remaining 2- and 3-ring PAHs with ILCRs lower than 10^−6^ are classified as lower cancer risk for humans, according to USEPA standards, where the acceptable risk of cancer from individual PAHs is (10^−6^ – 10^−4^).^53^

In addition, the risks from individual PAH exposure pathways were summarized to determine the key exposure pathway for each PAH compound contaminant surface soil in the study area. The results presented in Tables 3–5 clearly show a large difference between cancer risks for all 16 PAHs detected in soils via the inhalation route with a magnitude of (10^−13^~10^−10^ ), and the dermal contact and ingestion routes with a magnitude of (10^−8^~10^−6^) and (10^−9^~10^−6^), respectively. The results indicate that the dermal contact and ingestion pathways greatly contribute to the carcinogenic risk of human exposure to each PAH. Thus, higher dermal exposure contact can be explained by the emission of PAHs during traffic road, industrial and agricultural activities, which can be transferred to the surface of tools and clothing to the skin.^54^

In summary, the carcinogenicity of each PAH depends on its chemical structure and physical-chemical properties. The HMW PAHs are more potent carcinogens, and many studies have reported that exposure to HMW PAHs is linked to increased incidences of leukemia, bone, brain, bladder and scrotal cancers, and adverse pregnancy outcomes.^55^

### Cancer risk

In the present study, the cancer risk (R) of a total of 16 PAHs for children, adolescents, and adults had means of 2.48 × 10^−5^, 2.04 × 10^−5^, and 3.12 × 10^−5^ mg/kg/d, respectively (*Table 6*). These values are in the unacceptable range (10^−6^ ~10^−4^) an indicate a potential cancer risk to human health. In addition, the results showed that the ILCRs through the ingestion and dermal contact pathways ranged from 10^−7^ and 10^−5^ in soil samples, while the cancer risk through inhalation was 10^−12^ to 10^−9^, about 10^4^ to 10^5^ times lower than that through ingestion and dermal exposures. As shown in Table 6, the average values of total ILCRs calculated for the study area were similar to results found by Zha *et al.* (2018) in Nanjing, China.^10^

For children and adults, the cancer risk levels via dermal contact were in the same order of magnitude (10^−5^), showing that both dermal contact and ingestion had a significant contribution to the cancer risk in children and adults. Nevertheless, the risk of ingestion for adolescents (< 10^−6^) was lower than for children and adults, because children are more likely to be exposed to environmental pollutants and considered the most sensitive group age with frequent hand-to-mouth or object-to-mouth activities which facilitates ingestion of contaminated soils.^56^ For adults, a longer exposure time to the indoor/outdoor environment, greater body weight and greater skin surface area can also lead to a higher potential cancer risk.^57^ In the present study, the cancer risk for adults via dermal contact (1.99 × 10^−05^) was higher than for children and adolescents. This result is similar to the human cancer risk resulting from PAH exposure to surface soil in China cities (Beijing, Guangzhou, and Lanzhou), due to the larger dermal exposure with a long exposure period for adults.^34^ However, for adults, although the ingestion rate for soil (IRsoil) was relatively small, the longer exposure time may result in increased cancer risk, where can be similar to the risk of soil ingestion for children.

For adolescents, (13 to 20 years), the ILCRs for adolescents were lower due to lower soil ingestion rates and shorter exposure time. As shown in Table 3, the risk of cancer in children exposed to PAHs via dermal contact was significantly higher than that reported for adolescents. Furthermore, the risk of cancer in children from ingestion was equivalent to that of adults but higher than that of adolescents *(Table 4).*

Based on national and international regulatory agencies' delineated ILCR values, ≤ 10^−6^, 10^−6^ to 10^−4^, and >10^−4^ correspond to negligible risk, potential risk, and high risk of cancer, respectively.^14^,^58^ The ILCRs reported in the present study for children, adolescents, and adults due to PAH exposure (*Table 6*) were comparable with the ILCRs reported for various African locations like Warri city, Nigeria (children, 3.07 × 10^−5^; adults, 2.3 × 10^−5^),^59^ Kumasi city, Ghana (children, 8.5 × 10^−5^; adults 2.1 × 10^−4^),^60^ which indicate potential cancer risk. Values in the present study were lower than in Lagos, Nigeria (children, 6.66 × 10^−2^; adults, 5.11 × 10^−3^),^38^ Tamale, northern Ghana (children, 9.26 × 10^−1^; adults 1.02),^61^ dust from Warri city Nigeria (children, 3.11 × 10^−3^; adults, 1.49 × 10^−3^),^62^ and Delta state, Nigeria (children, 3.34 × 10^−1^; adults, 2.56 × 10^−2^),^63^ where a high risk of cancer was observed. Furthermore, the ILCRs were also comparable with those reported for locations in other countries such as Delhi-Kolkata, India (adults, 6.92 × 10^−5^ ; children 6.22 × 10^−5^)^3^ and Dhanbad, India (adults, 1.82 × 10^−5^; children 1.85 × 10^−5^).^64^ The present results were lower than values reported in Mahshahr, Iran (adults 1.20 × 10^−4^; children 1.56 × 10^−4^),^65^ Guangzhou, China (adults, 2.08 × 10^−4^ – 1.13 × 10^−2^; children, 2.26 × 10^−5^ – 1.23 × 10^−3^),^66^ and higher than in Kobra, India (adults, 5.6 × 10^−8^; children, 2.9 × 10^−7^).^40^

The R level for adults in the present study was higher than for children, while adolescents had the lowest risk *(Table 6).* Similar results have been reported in surface soils from the Tianjin coastal new region in China by Rongguang Shi *et al.* (2020),^67^ possibly due to fact that IR_soil_ and EF in children and adults were higher than in adolescents.

In addition, the R for children and adults in the study area was greater than the acceptable value of 1 × 10^−6^, indicating that the study area has the potential for human carcinogenic risk. Compared to other studies, the obtained results show that the total R values were in agreement with those reported in contaminated soils from Hong Kong metropolitan soils and urban soil from Nigeria.^59^,^67^

In general, preliminary health assessment results from the study area indicate that PAH-contaminated soils present a cancer risk to the resident population via cumulative ingestion and through dermal contact routes. The R for the three age groups in the present study indicate a potential health risk for adults and children, indicating that special attention should be paid to the health risks of children.

### Study limitations

Benarba *et al.* found a high incidence of liver cancer in the province, the highest in Algeria, suggesting a high prevalence of risk factors.^68^ Therefore, evaluation of PAH levels in soil and air, as well as other organic pollutants in specific sites with diverse activities (industrial, urban/residential) is recommended to better understand the potential effects on human health in this area.

## Conclusions

The present study represents the first health risk assessment from PAH-contaminated soils in northwestern Algeria. Total cancer risks of 16 PAHs for children, adolescents and adults were 2.48 × 10^−5^, 2.04 × 10^−5^ and 3.12 × 10^−5^ mg/kg/d. The risk of cancer for adolescents was within the acceptable range, with no potential risk of cancer, but there was a higher risk of moderate to severe cancer in children and adults in the study area. Among different exposure pathways, the observed cancer risks through dermal contact had the highest contribution to total cancer risks in the study area. Further research should focus on risk assessment of PAHs in specific areas as agricultural, residential/urban, and industrial, and should include exposure through air.
