# Supplementary material for: Human Health Assessment of Sixteen Priority Polycyclic Aromatic Hydrocarbons in Contaminated Soils of Northwestern Algeria
Source: J Health Pollut. 2021 Aug 17;11(31):210914. doi: 10.5696/2156-9614-11.31.210914 (PMC8383786; doi:10.5696/2156-9614-11.31.210914)
Supplement: Supplementary file 1 [file Halfadji_Supplemental_Material.docx]

**Supplemental Material**

**Table S1**: Chronic Daily Intake (CDI) (mg/kg/day) Across Age Groups to each Polyaromatic Hydrocarbon (PAH) in Contaminated Soils
via Different Exposure Pathways in the Study Area

|  | **CDIingestion** | | | **CDIinhalation** | | | **CDIdermal** | | |
| --- | --- | --- | --- | --- | --- | --- | --- | --- | --- |
|  | **CDIing** | **CDIing** | **CDIing** | **CDIinh** | **CDIinh** | **CDIinh** | **CDIderm** | **CDIderm** | **CDIderm** |
| **Individual PAH** | **Children** | **Adolescents** | **Adults** | **Children** | **Adolescents** | **Adults** | **Children** | **Adolescents** | **Adults** |
| Naphthalene | 1.45914 x 10^-8^ | 4.4405 x10^-09^ | 1.486 x10^-08^ | 5.3645 x10^-13^ | 5.2242 x10^-13^ | 1.74799 x10^-12^ | 5.31126 x10^-09^ | 6.5809 x10^-09^ | 7.7068 x10^-09^ |
| Acenaphthylene | 2.0485 x 10^-8^ | 6.2341 x10^-09^ | 2.086 x10^-08^ | 7.5312 x10^-13^ | 7.3342 x10^-13^ | 2.45402 x10^-12^ | 7.45653 x10^-09^ | 9.2389 x10^-09^ | 1.082 x10^-08^ |
| Acenaphthene | 4.10583 x 10^-09^ | 1.2495 x10^-09^ | 4.181 x10^-09^ | 1.5095 x10^-13^ | 1.47 x10^-13^ | 4.91863 x10^-13^ | 1.49452 x10^-09^ | 1.8518 x10^-09^ | 2.1686 x10^-09^ |
| Fluorene | 4.11788 x 10^-09^ | 1.2532 x10^-09^ | 4.193 x10^-09^ | 1.5139 x10^-13^ | 1.4743 x10^-13^ | 4.93307 x10^-13^ | 1.49891 x10-^09^ | 1.8572 x10^-09^ | 2.175 x10^-09^ |
| Phenanthrene | 7.59579 x 10^-08^ | 2.3116 x10^-08^ | 7.735 x10^-08^ | 2.7926 x10^-12^ | 2.7195 x10^-12^ | 9.09946 x10^-12^ | 2.76487 x10^-08^ | 3.4258 x10^-08^ | 4.0119 x10^-08^ |
| Anthracene | 3.1308 x10^-08^ | 9.5278 x10^-09^ | 3.188 x10^-08^ | 1.151 x10^-12^ | 1.1209 x10^-12^ | 3.75058 x10^-12^ | 1.13961 x10^-08^ | 1.412 x10^-08^ | 1.6536 x10^-08^ |
| Fluoranthene | 3.12425 x10^-07^ | 9.5079 x10^-08^ | 3.181 x10^-07^ | 1.1486 x10^-11^ | 1.1186 x10^-11^ | 3.74273 x10^-11^ | 1.13723 x10^-07^ | 1.4091 x10^-07^ | 1.6502 x10^-07^ |
| Pyrene | 3.12553 x10^-07^ | 9.5118 x10^-08^ | 3.183 x10^-07^ | 1.1491 x10^-11^ | 1.119 x10^-11^ | 3.74427 x10^-11^ | 1.13769 x10^-07^ | 1.4097 x10^-07^ | 1.6508 x10^-07^ |
| Benzo[a]anthracene | 2.28111 x10^-07^ | 6.942 x10^-08^ | 2.323 x10^-07^ | 8.3864 x10^-12^ | 8.1671 x10^-12^ | 2.73268 x10^-11^ | 8.30323 x10^-08^ | 1.0288 x10^-07^ | 1.2048 x10^-07^ |
| Chrysene | 6.48456 x10^-08^ | 1.9734 x10^-08^ | 6.603 x10^-08^ | 2.384 x10^-12^ | 2.3217 x10^-12^ | 7.76826 x10^-12^ | 2.36038 x10^-08^ | 2.9246 x10^-08^ | 3.425 x10^-08^ |
| Benzo[b]fluoranthene | 1.2982 x10^-07^ | 3.9508 x10^-08^ | 1.322 x10^-07^ | 4.7728 x10^-12^ | 4.6479 x10^-12^ | 1.55519 x10^-11^ | 4.72544 x10^-08^ | 5.855 x10^-08^ | 6.8568 x10^-08^ |
| Benzo[k]fluoranthene | 6.92086 x10^-08^ | 2.1062 x10^-08^ | 7.047 x10^-08^ | 2.5444 x10^-12^ | 2.4779 x10^-12^ | 8.29092 x10^-12^ | 2.51919 x10^-08^ | 3.1214 x10^-08^ | 3.6554 x10^-08^ |
| Benzo[a]pyrene (BaP) | 8.69697 x10^-08^ | 2.6467 x10^-08^ | 8.856 x10^-08^ | 3.1974 x10^-12^ | 3.1138 x10^-12^ | 1.04186 x10^-11^ | 3.1657 x10^-08^ | 3.9224 x10^-08^ | 4.5935 x10^-08^ |
| Indeno(1,2,3-c,d)pyrene | 7.05865 x10^-08^ | 2.1481 x10^-08^ | 7.188 x10^-08^ | 2.5951 x10^-12^ | 2.5272 x10^-12^ | 8.456 x10^-12^ | 2.56935 x10^-08^ | 3.1835 x10^-08^ | 3.7282 x10^-08^ |
| Dibenzo(a,h)anthracene | 1.7271 x10^-08^ | 5.256 x10^-09^ | 1.759 x10^-08^ | 6.3496 x10^-13^ | 6.1835 x10^-13^ | 2.069 x10^-12^ | 6.28665 x10^-09^ | 7.7894 x10^-09^ | 9.1221 x10^-09^ |
| Benzo(g,h,i)perylene | 6.74891 x10^-08^ | 2.0539 x10^-08^ | 6.872 x10^-08^ | 2.4812 x10^-12^ | 2.4163 x10^-12^ | 8.08494 x10^-12^ | 2.4566 x10^-08^ | 3.0438 x10^-08^ | 3.5646 x10^-08^ |
| **∑2-3-ring** | 1.50566 x10^-07^ | 4.5821 x10^-08^ | 1.533 x10^-07^ | 5.5355 x10^-12^ | 5.3907 x10^-12^ | 1.80372 x10^-11^ | 5.4806 x10^-08^ | 6.7907 x10^-08^ | 7.9525 x10^-08^ |
| **∑4ring** | 9.17934 x10^-07^ | 2.7935 x10^-07^ | 9.347 x10^-07^ | 3.3748 x10^-11^ | 3.2865 x10-^11^ | 1.09965 x10^-10^ | 3.34128 x10^-07^ | 4.14 x10^-07^ | 4.8483 x10^-07^ |
| **∑5ring** | 3.03269 x10^-07^ | 9.2293 x10^-08^ | 3.088 x10^-07^ | 1.115 x10^-11^ | 1.0858 x10^-11^ | 3.63305 x10^-11^ | 1.1039 x10^-07^ | 1.3678 x10^-07^ | 1.6018 x10^-07^ |
| **∑6ring** | 1.38076 x10^-07^ | 4.202 x10^-08^ | 1.406 x10^-07^ | 5.0763 x10^-12^ | 4.9435 x10^-12^ | 1.65409 x10^-11^ | 5.02595 x10^-08^ | 6.2274 x10^-08^ | 7.2928 x10^-08^ |
| **∑LMW-PAHs** | **1.50566 x10^-07^** | **4.5821 x10^-08^** | **1.53 x10^-07^** | **5.5355 x10^-12^** | **5.39 x10^-12^** | **1.80372 x10^-11^** | **5.4806 x10^-08^** | **6.7907 x10^-08^** | **7.9525 x10^-08^** |
| **∑HMW- PAHs** | **1.35928 x10^-06^** | **4.1366 x10^-07^** | **1.384 x10^-06^** | **4.9973 x10^-11^** | **4.8666 x10^-11^** | **1.62836 x10^-10^** | **4.94778 x10^-07^** | **6.1305 x10^-07^** | **7.1794 x10^-07^** |
